# Treatment of sheep prior to movement: its contribution to an effective scab (psoroptic mange) management strategy

**DOI:** 10.1186/s13071-023-06044-0

**Published:** 2023-11-26

**Authors:** Katie Lihou, Richard Wall, Emily Nixon

**Affiliations:** 1 Department of Engineering Mathematics, University of Bristol, Bristol, UK; 2 School of Biological Sciences, University of Bristol, Bristol, UK; 3 Department of Mathematical Sciences, University of Liverpool, Liverpool, UK

**Keywords:** Ovine psoroptic mange, Sheep scab, Epidemiological modelling, Disease control

## Abstract

**Background:**

Ovine psoroptic mange (sheep scab) is an important disease of sheep worldwide caused by the parasitic mite, *Psoroptes ovis*. It has a negative impact on animal welfare and leads to significant economic losses for the sheep industry. Effective and targeted management is required to limit its transmission.

**Methods:**

A stochastic metapopulation model of sheep scab transmission is used to investigate the contribution of the treatment of sheep prior to movements to sales, gatherings (predominantly markets) and away grazing to the reduction of prevalence of farms with scab in Great Britain.

**Results:**

Treatment prior to movement to gatherings resulted in an 86% reduction in the overall prevalence of farms with scab and was more effective at reducing the overall prevalence of farms with scab than treatment before other categories of movements. The relative risk of farms having scab infection was inversely related to the percentage of farms which treated, but this relationship was not linear, with the biggest declines in the prevalence of farms with scab being achieved by small percentages of farms treating; a 50% relative reduction in the farm prevalence was achieved with only 15% of farms treating prior to gathering movements.

**Conclusions:**

The results suggest that pre-movement treatment of sheep could make an important contribution to national scab control and, in practice, the approach could be more highly targeted if used in conjunction with known geographic and management risk factors for scab.

**Graphical Abstract:**

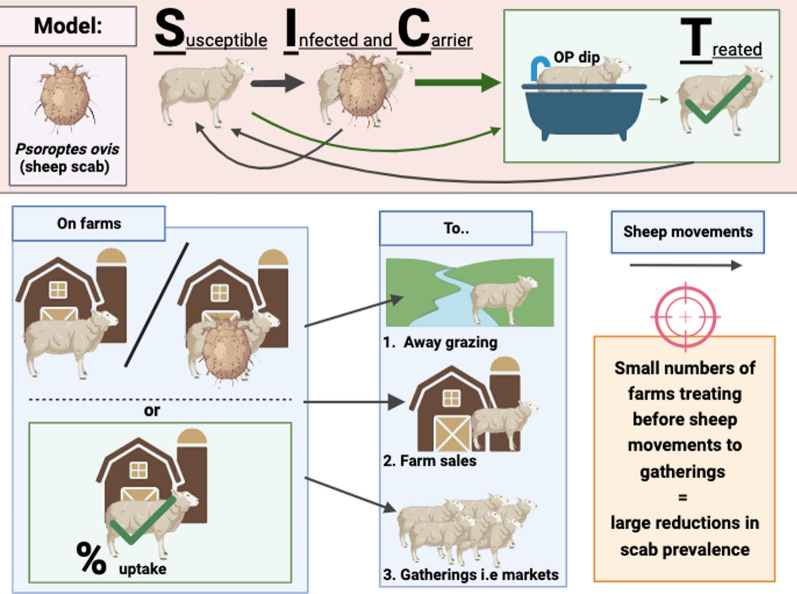

## Background

Ovine psoroptic manage (sheep scab), caused by the mite *Psoroptes ovis*, is not only a welfare concern but also has significant economic impacts for the farming industry [1]. Many different approaches, often centred around increasing farmer awareness and education, have been used in an attempt to reduce the incidence of sheep scab in Great Britain; however, to date, none of these have prevented the increase in annual scab incidence since deregulation in 1992 [2].

Recent attempts to use mathematical modelling to identify the key points in disease epidemiology where intervention might be most effective have been applied effectively to diseases such as foot and mouth [3], bovine tuberculosis [4] and avian influenza [5]. A mathematical model for sheep scab has been developed [6] and has highlighted the likelihood that spatial clusters of contiguous farms exist, between which local transmission of scab occurs by contact between sheep with a shared contaminated environment, as suggested by French et al. [7]. At the boundaries of these clusters, where the distances between farms is greater, transmission rates are likely to be low, and scab would self-limit, if farm-to-farm contact was the only transmission route [8]. These clusters correspond geographically with areas that have been identified previously as having a higher scab prevalence and risk compared to other areas in Great Britain [9]. It has been suggested that focusing control within these spatial clusters may be a cost-effective means to control scab [8]. The fact that scab does not self-limit in these regions supports the observation that the movement of infected animals between areas is also an important transmission route [10].

The movement of infected sheep between regions would be expected to undermine local management and these movements include direct farm-to-farm sales, sales through markets and seasonal away grazing. Hence, the treatment of sheep prior to movement may prevent long distance spread out of high-risk areas, therefore reducing overall levels of scab infection and making localised management more effective. However, it is unclear whether all animals would need to be treated, whether treatment at particular times of year are more likely to be effective than treatment at other times, whether the treatment of animals undertaking movement for different reasons has a greater or lesser effect and, finally, whether there is a critical threshold of the number of farms that would need to treat to optimise any effect. As a result, the work presented here aimed to use a modified version of an existing sheep scab model to explore a range of pre-movement treatment strategies to assess whether this form of targeted treatment could be effective in reducing sheep scab infection in the absence of other management approaches.

## Methods

The model used in this study was an existing open access stochastic metapopulation model for sheep scab transmission [5, 7, 10]. The model was built and run in R v.4.2.2 [12] based on a modified version of the R package “SimInf” v.9.5.0 [13]. The model was then also modified to include the prophylactic treatment of sheep for scab prior to sheep movements.

The model includes all georeferenced sheep holdings in Great Britain and allows transmission to occur within and between holdings. The spatial coordinates, numbers of sheep at each sheep holding and movements of sheep between holdings in Great Britain in 2010 were obtained from the Animal and Plant Health Agency (APHA) of the UK government. These were extracts from the June Census of Agriculture and Horticulture for England and Wales, the June Agricultural Census for Scotland, the Animal Movement Licensing System for England and Wales and the Scottish Animal Movement System for Scotland. Contiguous farms were then identified by using the easting and northing data to calculate the Euclidean distance between centres of farms using the distance matrix function from the “SimInf” package [13].

Transmission of scab within and between contiguous holdings was modelled using a stochastic metapopulation model, with each sheep holding as a subpopulation in the model. Within each holding, sheep are classified into compartments: susceptible (S), infected (I) and carrier (C). The number of susceptible sheep that become infected is determined by an infectious pressure exerted by a compartment (*e*) which is contingent on the transmission and shedding of *Psoroptes ovis* mites from infectious sheep either to the environment or directly to in-contact sheep within a holding or contiguous holdings.

The model also allowed the transmission of scab between holdings to occur via the movements of infected sheep, which are specified as scheduled deterministic events executed when the simulation (in continuous time) reaches the specified timestep for the event. The specified number of sheep to be moved is sampled at random across all disease compartments in the source holding and then transferred to the corresponding disease compartment in the destination holding. Sheep movement data for 2010 were provided by APHA and were used to specify the deterministic movement events for given numbers of sheep between specific source and destination holdings on specific dates. It was assumed that all holdings which were temporary residences, such as markets, had no sheep at the start of each simulation.

The parameters *α*, the daily contribution to environmental pressure per infected individual, *β*, the decay rate of the environmental infectious pressure and *υj*, the indirect transmission rate from the environmental compartment (*j*) to susceptible sheep in farm *i* were estimated in a previous study which used sequential Monte Carlo approximate Bayesian Computation (SMC ABC) methods to fit the model to weekly and yearly scab incidence cases from 1973 to 1992 [6]. The upper ranges of the posterior distribution from the SMC ABC for the two transmission rates (*α _* = 1 × 102, *υj* = 6 × 104) and the lower range of the posterior distribution for the disease decay rate (*β _* = 4 × 102) were used in the current study to allow for transmission patterns across the network to be observed. Other parameters were determined using published data (as described in [6]). All scenarios were run with 30 stochastic repeats.

### Initial infection

The simulations were all run with an initial national scab prevalence of 9% of farms infected based on data collected from farmer questionnaire surveys and on publicly available surveillance data [14]. Previous modelling work found no discernible difference in quantitative results between scenarios where initial scab prevalence was 3% or 9% [11], so the results presented here should be applicable to a range of realistic national scab prevalence starting values. The county level prevalence was then scaled based on county prevalence estimated from survey data [15]. Each scenario was run 30 times, each with a different combination of randomly generated initially infected farms.

### Scenarios

A scenario with no treatments administered against sheep scab was used as a baseline. Treatment was modelled as a scheduled event, where a stated proportion of sheep within specified holdings are pre-determined to move from the S, I and C compartments to a “Treated” (T) compartment on a particular timestep in the simulation. All simulations were run for a 1-year period starting on January 1st.

All treatment scenarios assumed that treatment occurred the day before the sheep movement, that all treatments were conducted with an organophosphate (OP) full-immersion dip which had a residual protection of 60 days (Veterinary Medicines Directorate, 2009) and that treatment efficacy was 98% to account for misapplication. At the end of the period of residual activity all sheep from the treatment compartment are moved back to the susceptible compartment. The treatment scenarios modelled were treatments prior to sheep movement for the purpose of: (1) gatherings, (2) sales and (3) away grazing.

The category ‘gatherings’ includes sheep moved for sale through markets, shows and breed inspections. Sales include direct movements from one holding to another, and ‘away grazing’ includes movement to alternative pasture, most often overwinter, and was characterised by the subsequent return of animals to their original holding. Common grazing movements were not included as these are generally short distance movements which were not recorded in data available. Sheep movements to slaughter premises were also not included as these sheep will not contribute to the onward transmission of scab.

For each of these scenarios it was initially assumed that all farmers treated their sheep prior to the movement (acaricide application does not require prescription or veterinary intervention). To test how the uptake of treatment strategies by different proportions of farmers might impact the overall effectiveness of the strategy, model simulations were run for 10%, 25%, 40%, 50%, 60%, 75% and 100% of farmers treating prior to movement to gatherings. Each percentage scenario included five stochastic repeats of combinations of farms which treated; then each of these five combinations was repeated with six combinations of initially infected farms, so each percentage scenario was made up of 30 stochastic repeats. Model scenario outputs were fitted to a general additive model (GAM) using the “mgcv” package with ‘family = inverse.gaussian’ in R and used to predict the average prevalence of farms with scab for every percentage increase in the percentage of farms treating. The value of k, which affects the degree of smoothness, was selected based on the value in which the k-index was closest to 1 [16]. Assumptions for a GAM model were checked using the ‘gam.check’ function.

All scenario results were evaluated as the mean daily prevalence of farms with scab. Scenarios are presented in the form of relative risk, comparing average prevalence to a no treatment baseline scenario (number of farms with scab in treatment scenario/number of farms with scab in baseline scenario), where zero represents no farms with scab to allow the relative effectiveness of each treatment scenario to be assessed.

## Results

### Movement data

**Fig. 1:**
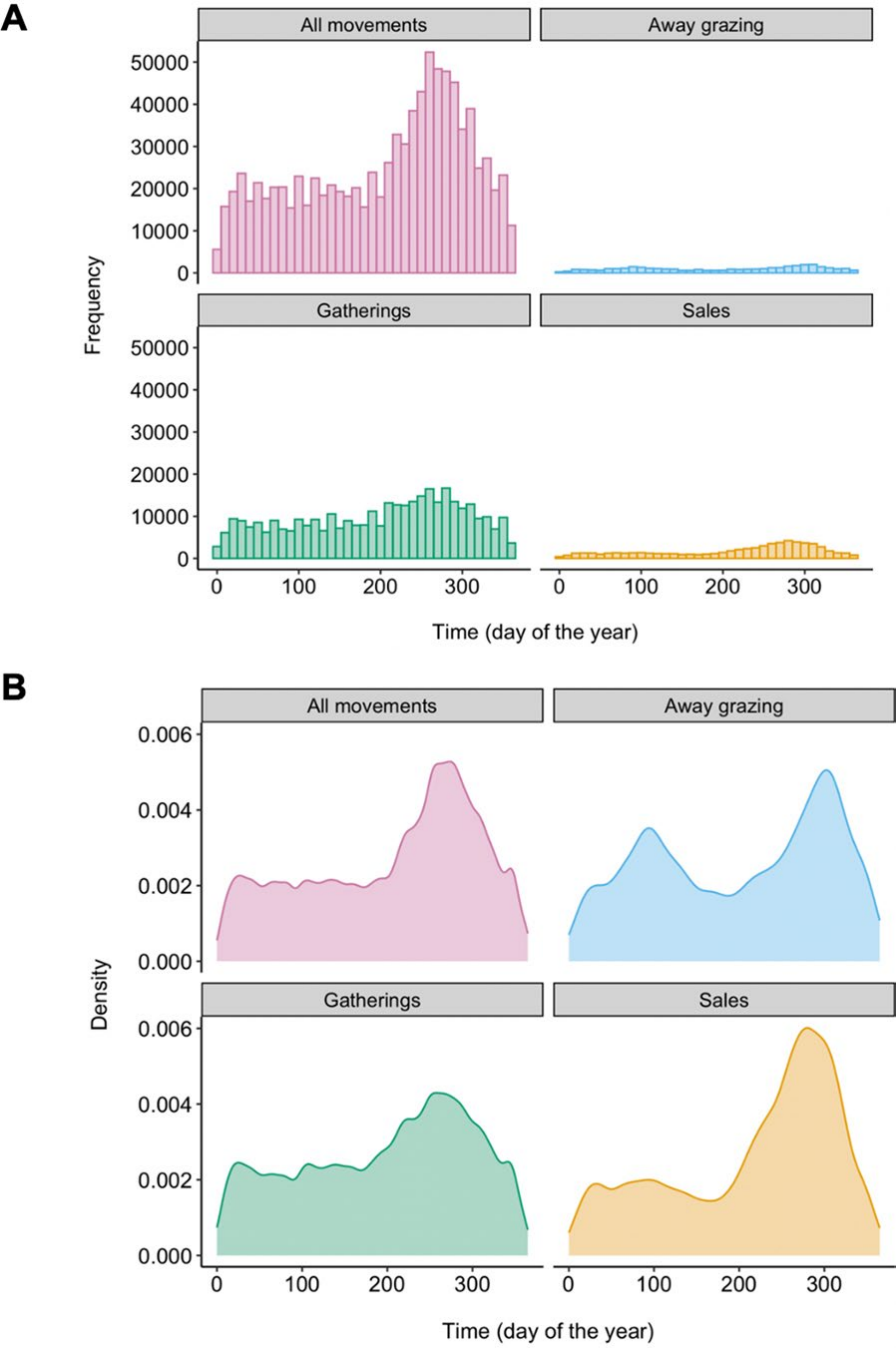
**A** The number (frequency) of recorded sheep movement events over the year (2010) for different types of movement destinations. **B** The smoothed kernel density estimate of sheep movement events over the year (2010), for different types of movement destinations

In the dataset used in the model, sheep movement events to gatherings were more frequent than movement events to other holdings for the purpose of away grazing or sales (Fig. 1a). The number of sheep movement events showed a peak in the autumn, and this was consistent across all movement event types examined (Fig. 1b). This peak in movement events in the autumn was mirrored by a rise in the model output of the prevalence of farms with scab at this time of year [11].

**Fig. 2:**
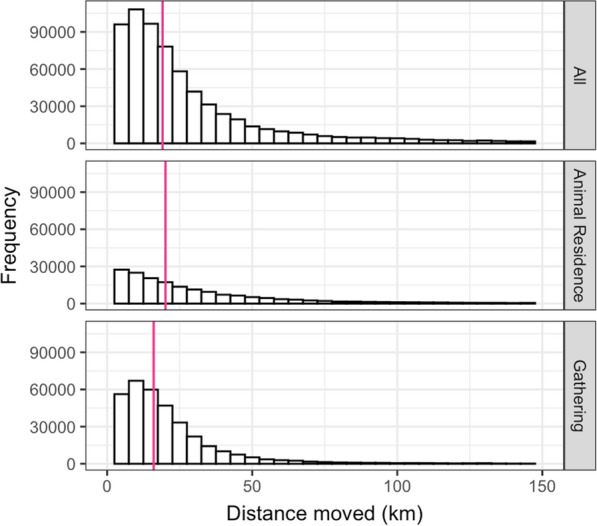
The number (frequency) of recorded sheep movement events over the year (2010) for different movement distances (up to 150 km). Histograms are shown for different types of movement destinations: ‘All’, ‘Animal residence’ and ‘Gathering’. Pink lines show the median movement distance for each destination type

The median distance for all movement events was 19.1 km (IQR = 27.0 km; Fig. 2). The median distance was 20.1 km (IQR = 33.8 km) for sheep movement events to sales and away grazing and 16.0 km (IQR = 17.0 km) for sheep movement to gatherings.

### Treatment scenarios

**Fig. 3:**
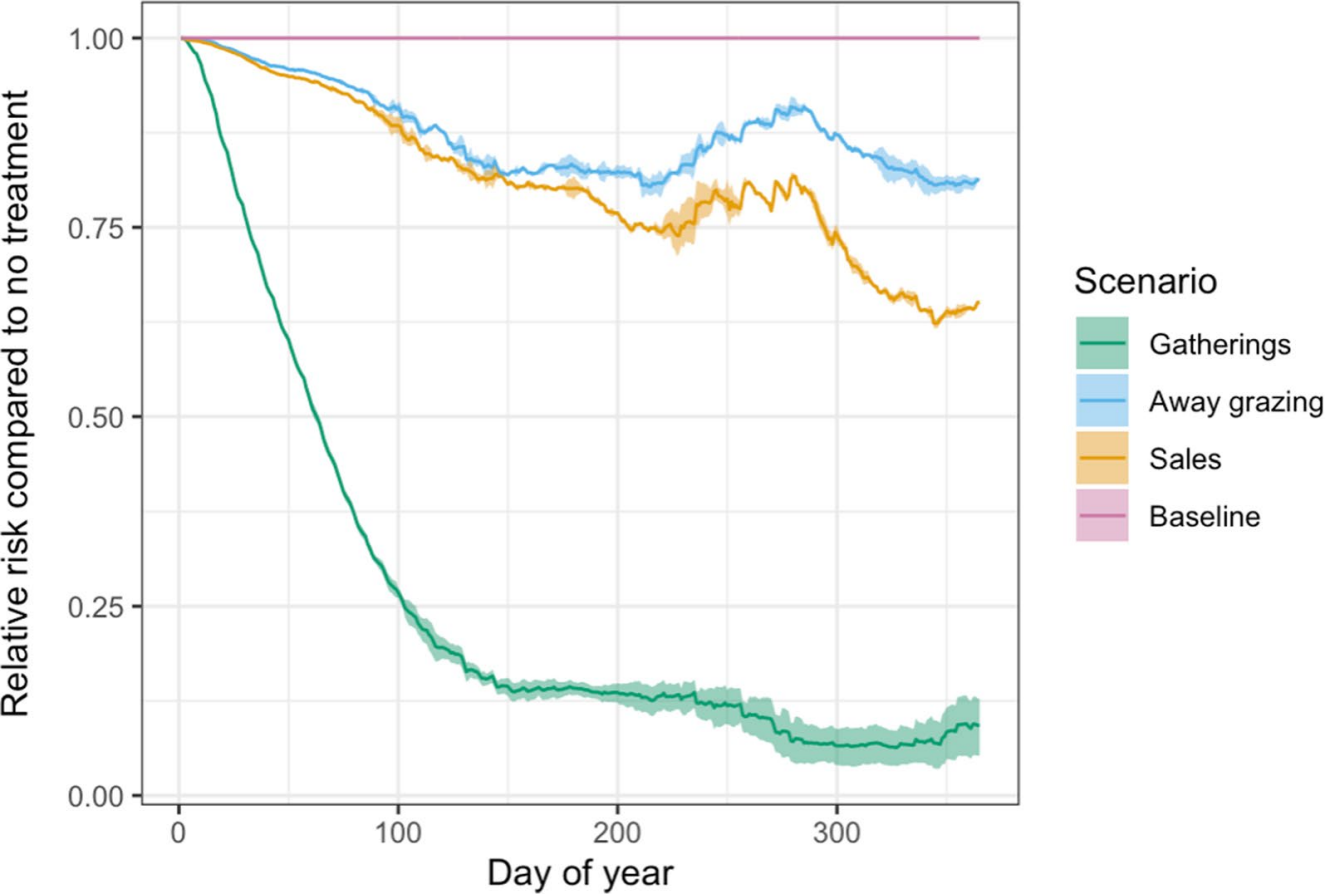
The relative risk of farms having a sheep scab infestation for each day of the year relative to a no-treatment baseline for different pre-movement treatment scenarios where 100% of farms treat their sheep prior to each movement

Treatment prior to gatherings had a bigger impact on the daily prevalence of farms with scab than either movements for away grazing or sales between holdings (Fig. 3). The median daily risk reduction was 86.2% (IQR = 21.1%) for pre-gathering treatments, 19.8% (IQR = 14.6%) for pre-sales treatments and 13.9% (IQR = 8.6%) for pre-away grazing treatments. The reduction in the prevalence of farms with scab in the pre-gathering treatment scenario relative to other scenarios generally increased throughout the year and was most pronounced in the autumn months (Fig. 3).

### Treatment uptake threshold

**Fig. 4:**
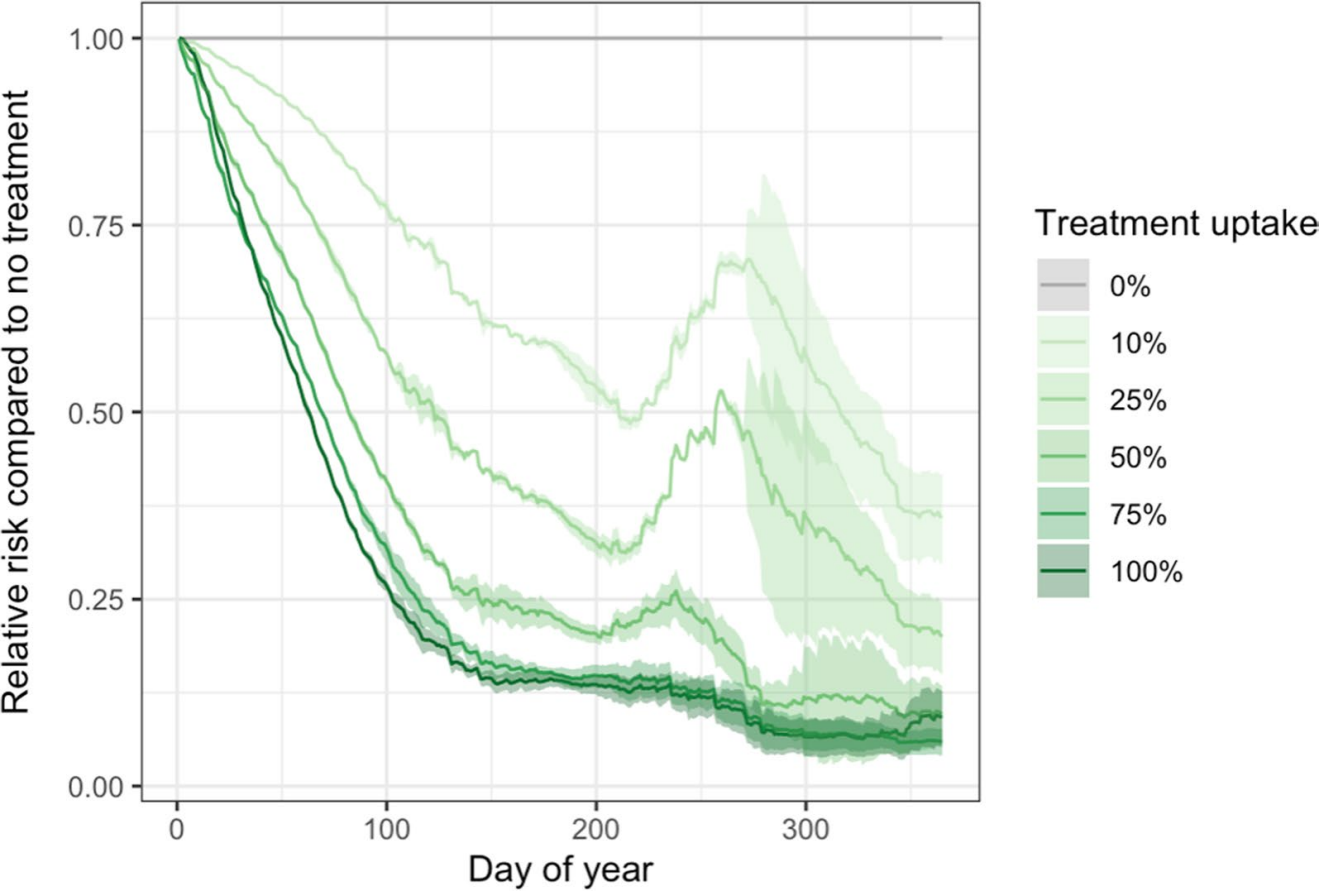
The relative risk of farms having a sheep scab infestation for each day of the year relative to a no-treatment baseline for different pre-gathering treatment scenarios where different percentages of farmers treat (10%, 25%, 50%, 75% and 100%)

The daily risk of farms having scab infection relative to a no treatment scenario was reduced as higher percentages of farms treated their sheep prior to movements to gatherings, and a reduction in relative risk was achieved by only 10% of farms treating (Fig. 4). The daily relative risk of farms having scab infection in the scenarios where 10%, 25% or 50% of farms treated was higher in autumn than in the surrounding months. There was little difference in the relative risk of farms having scab between 75% and 100% of farms treating and these scenarios were the most effective at reducing the prevalence of farms with scab across the year relative to other scenarios and especially in the autumn months.

**Fig. 5:**
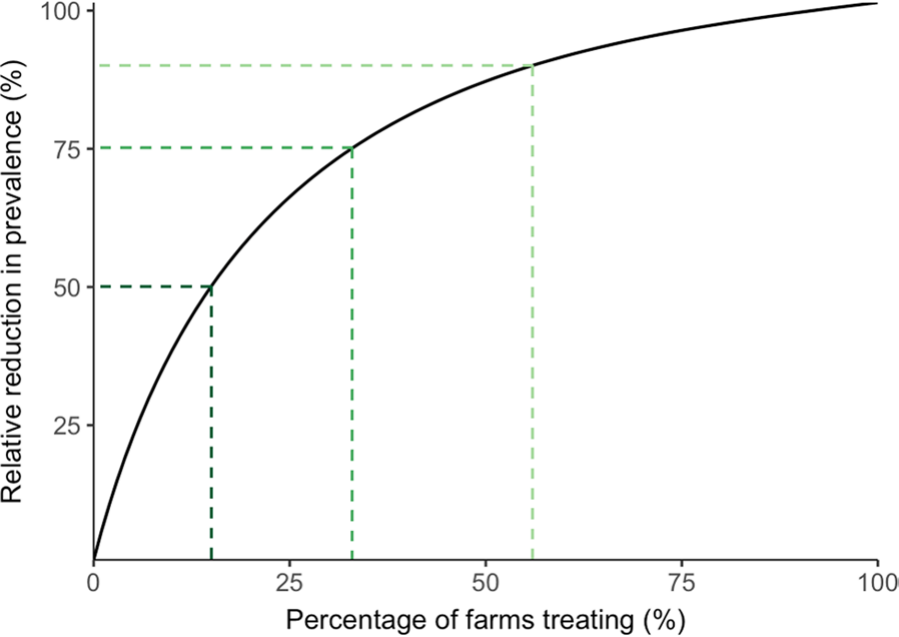
The relative reduction in the overall prevalence of farms with sheep scab with different percentages of farmers treating their sheep prior to gathering movements. The upper and lower bounds of prevalence were taken as the prevalence when 0% and 100% of farms treated, respectively. The prevalence of farms with sheep scab for each percentage of farmers treating was predicted from a fitted GAM model and plotted as a curve. The dashed lines show the percentage of farmers that need to treat to reduce relative prevalence by 50% (dark green), 75% (medium green) and 90% (light green)

Model scenario outputs were fitted to a general additive model and the average daily prevalence of farms with scab was predicted for every percentage increase in the percentage of farms treating. As the percentage of farms treating increased, the relative reduction in prevalence of farms with scab increased rapidly at first and then more slowly (Fig. 5). To reduce relative scab prevalence by 50%, only 15% of farms needed to treat prior to gathering movements. To reduce relative scab prevalence by 75%, 33% of farms needed to treat, and to reduce relative scab prevalence by 90%, 56% of farms needed to treat (Fig. 5).

## Discussion

Previous modelling has suggested that ‘National Movement Control’, where all sheep are treated prior to movement, is likely to contribute effectively to reducing scab transmission to almost zero [11]. However, this strategy would be treatment-intensive, and hence costly and hard to enforce, and is potentially environmentally damaging [17]. The aim of the analysis reported here was to refine this scenario to investigate whether treatments prior to sheep movements could be targeted to reduce insecticide use while still controlling scab transmission. The analysis demonstrated that treatment prior to gatherings resulted in an 86% reduction in the relative risk of farms having scab and was more effective at reducing the overall prevalence of farms with scab than treatment prior to movement to other animal holdings for sales or away grazing. This will be partly because the relative proportion of movements to gatherings in the dataset is higher than movements to animal residences (Fig. 1a), but in addition, gatherings represent larger collections of sheep in single locations compared to other types of movement, so the transmission potential within these types of holdings will be higher. In the model, when calculating the rate of transmission, the area of a holding is assumed to be proportional to the number of individuals in each holding. While this is generally a reasonable assumption for most sheep holdings, in gatherings such as markets, a higher number of sheep may be kept together in a smaller area than they would usually, increasing the transmission potential. Therefore, treating prior to moving sheep to gatherings may be even more important than the model suggests, and future work could adapt the model to explore more about transmission between pens at gatherings using a similar approach to Tuominen et al. [18].

The model results showed that the larger the percentage of farms that treated prior to moving their sheep to gatherings, the lower the overall prevalence of farms with scab (Fig. 4). However, this relationship was not linear, with the biggest declines in scab prevalence being achieved by small percentages of farms treating (Fig. 5); a 50% reduction in prevalence was achieved by only 15% of farms treating, suggesting that any percentage of treatment uptake by farmers is desirable in reducing overall transmission. There was no specific threshold above which there was no further reduction in overall farm prevalence, but to achieve a 90% reduction in overall farm prevalence only 56% of farms had to treat. Although not investigated here, it is likely that targeting the treatment to farms moving high volumes of livestock, and those with a high-risk of scab, would even further improve the efficacy of this strategy. Known risk factors, such as geographic location, the use of common grazing or scab history, could be used as indicators of risk. Therefore, if the aim of treatment is to reduce transmission rather than eradicate the disease, a national scab treatment programme could target pre-movement treatments to and from high-risk farms, which would minimise the environmental impacts associated with acaricide use [17].

The scenario results showed seasonal variation in the effectiveness of treatments. All treatment scenarios showed a sharp decline in relative risk during the first part of the year caused by the initial effects of treatment on scab transmission combined with seasonal effects. The model scenarios presented here were all run for 1 year only, but if they were continued beyond the first year, the initial reductions in farm prevalence caused by the initial treatments would have an accumulative effect, and may result in further prevalence declines over time, before reaching endemic stability. The pre-gathering percentage treatment uptake scenarios showed more uncertainty in the autumn. This is most likely to be caused by the peak in sheep movements at this time (Fig. 1), which correlates with a spike in scab prevalence [11]. The magnitude of this spike will vary depending on the proportion of infected sheep movements which are treated. This highlights that effectively targeting treatment can have a big impact on the efficacy of the treatment strategy, and this is especially important at times of year where many of sheep movements occur, such as in the autumn.

The median movement distance was 19 km (Fig. 2), which is much higher than the 2 km distance which was defined as the maximum distance for contiguous holdings—where local transmission of scab can occur through contact of sheep with a shared contaminated environment [8]. This supports the theory that long-distance movements are important in transmitting scab between regions and could destabilise regional control strategies. Targeted pre-movement treatments could be combined with regional control strategies in high-risk areas, as previously modelled [11], to prevent regional treatments from being undermined by the importation of infected sheep from further afield.

An assumption of the model is that all treatments used are organophosphate (OP) dips with an efficacy of 98% to account for misapplication and residual activity over 60 days after application. However, not all sheep owners may choose to use an OP dip, as endectocides [injectable group 3-macrocycline lactones (ML)] are also licensed treatments. Most, though not all, ML products have shorter periods of residual activity. Furthermore, while there have been no reports of resistance in *P. ovis* against OP dips, resistance to MLs has been reported [19, 20] and therefore treatment efficacy in the model may be overestimated. When planning future control programmes for scab, it will continue to be important to emphasise to farmers the risk of treatment failure and the importance of correct treatment application.

In the Animal Movement Licensing System (AMLS) for England and Wales, movements of sheep to graze on common land do not need to be recorded when the common grazing land borders the sheep holding. In addition, movements within an 8 (Wales) or 16 (England) km (5–10 mile) radius of a sheep holding do not need to be recorded. In the Scottish Animal Movement System (SAMS) for Scotland, all movements, including common grazing movements, must be recorded, unless the move is to common grazing in a crofting township [21]. As only the movements that were recorded in AMLS and SAMS were used in the model, any movements that were not captured in the data will not have been modelled. This may have led to an underestimation of transmission that occurs during grazing on common land in England and Wales and in crofting townships in Scotland. If data become available relating to these unrecorded movements, future work could include this in the simulation to investigate the impact of treatment prior to sending sheep to common grazing. The model used national sheep movement data from 2010, since this was available to the authors; no major changes in national movement patterns in the intervening years are known; nevertheless, this is an assumption of the study, and future work should seek to use updated movement data.

## Conclusions

Overall, this work demonstrates that future control programmes for scab should focus on encouraging treatment prior to movement to gatherings, which could lead to a 50% relative reduction in scab prevalence across farms in Great Britain even if only 15% of farmers adopt this strategy. The approach could be even more tightly targeted if treatment prior to movement to gatherings was used in conjunction with known scab risk factors.

## Data Availability

The individual farm and animal movement data that support the findings of this study were provided by the Animal and Plant Health Agency of the UK Government, but restrictions apply to the availability of these data, which were used under license for the current study, and so are not publicly available from the authors. The model code is available on GitHub at https://github.com/emjnixon15/ScabModel/tree/Movement_paper, along with the data outputs from the simulations.
